# Cortical Seizures in *FoxG1^+/−^* Mice are Accompanied by Akt/S6 Overactivation, Excitation/Inhibition Imbalance and Impaired Synaptic Transmission

**DOI:** 10.3390/ijms20174127

**Published:** 2019-08-24

**Authors:** Giovanna Testa, Francesco Olimpico, Laura Pancrazi, Ugo Borello, Antonino Cattaneo, Matteo Caleo, Mario Costa, Marco Mainardi

**Affiliations:** 1 Laboratory of Biology “Bio@SNS”, Scuola Normale Superiore, 56126 Pisa, Italy; 2 Institute of Neuroscience, Italian National Research Council (IN-CNR), 56124 Pisa, Italy; 3 Unit of Cell and Developmental Biology, Department of Biology, University of Pisa, 56123 Pisa, Italy; 4 European Brain Research Institute “Rita Levi-Montalcini” (EBRI), 00161 Rome, Italy; 5 Department of Biomedical Sciences, University of Padua, 35131 Padua, Italy

**Keywords:** epilepsy, Rett syndrome, electrophysiology

## Abstract

The correct morphofunctional shaping of the cerebral cortex requires a continuous interaction between intrinsic (genes/molecules expressed within the tissue) and extrinsic (e.g., neural activity) factors at all developmental stages. Forkhead Box G1 (FOXG1) is an evolutionarily conserved transcription factor, essential for the cerebral cortex patterning and layering. FOXG1-related disorders, including the congenital form of Rett syndrome, can be caused by deletions, intragenic mutations or duplications. These genetic alterations are associated with a complex phenotypic spectrum, spanning from intellectual disability, microcephaly, to autistic features, and epilepsy. We investigated the functional correlates of dysregulated gene expression by performing electrophysiological assays on *FoxG1^+/−^* mice. Local Field Potential (LFP) recordings on freely moving animals detected cortical hyperexcitability. On the other hand, patch-clamp recordings showed a downregulation of spontaneous glutamatergic transmission. These findings were accompanied by overactivation of Akt/S6 signaling. Furthermore, the expression of vesicular glutamate transporter 2 (vGluT2) was increased, whereas the level of the potassium/chloride cotransporter KCC2 was reduced, thus indicating a higher excitation/inhibition ratio. Our findings provide evidence that altered expression of a key gene for cortical development can result in specific alterations in neural circuit function at the macro- and micro-scale, along with dysregulated intracellular signaling and expression of proteins controlling circuit excitability.

## 1. Introduction

The complexity of the mature six-layered structure of the mammalian cerebral cortex is achieved through a long and precisely regulated developmental process controlling neurogenesis, neuronal migration and differentiation. Cortical abnormalities caused by altered expression of genes regulating brain development at different levels, e.g., gene transcription [1] or cell migration [2], constitute a significant fraction of pediatric pathologies associated to intellectual disability [3]. Among these, homozygous mutations in *FoxG1* lead to perinatal lethality in mice [4], in line with the role of this transcription factor in orchestrating telencephalic development [5]. On the other hand, heterozygous mutations in *FOXG1* are compatible with life, but result in reduced size of cerebral hemispheres, alterations in cortical layering [6] and severe intellectual disability with autism spectrum disorder (ASD)-like features [7]. From a clinical point of view, the phenotype associated with mutations in *FOXG1* constitutes a significant fraction of Rett syndrome (RTT) cases (OMIM 613454) [8]. In comparison to “classical” RTT due to *MECP2* mutations, *FoxG1*-associated variants share ASD signs, but also tend to show an earlier onset, a higher occurrence of epileptic seizures, and callosal abnormalities [9]. In addition, the localization of *FoxG1* on the 14q12 chromosomal region allows the mutation to cause the pathology in both sexes, in contrast to X-linked RTT caused by *MECP2* mutations [7].

*FOXG1*-associated RTT is mostly due to deletions [7]; consistently, the cardinal signs of this neurodevelopmental pathology are reproduced in *FoxG1^+/−^* mice harboring a disrupted allele of the gene, including reduced cortical volume and cognitive impairment [4,10].

Among the abnormalities contributing to the RTT phenotype, epilepsy gives a significant negative contribution to the patients’ quality of life. Thus, a better understanding of the details of this comorbidity, from both functional and biochemical-molecular points of view, could be exploited to instruct the elaboration of more effective therapies. In this regard, we recently showed that *FoxG1^+/−^* mice display hippocampal epileptiform electrophysiological events and higher propensity to proconvulsant-induced generalized seizures, in comparison to wild type controls [11]. However, the cortical in vivo electrophysiological phenotype of *FoxG1^+/−^* mice has only been studied incompletely. Indeed, visual evoked potential recordings under anesthesia demonstrated reduced visual acuity, despite a normal retinal structure, which can be related to the deficit in face recognition and mismatch between looking and reaching observed in juvenile *FOXG1*-haploinsufficient patients [12]. These findings prompted us to better study cortical electrophysiology in freely moving *FoxG1^+/−^* mice and, using local field potential recordings with chronically implanted electrodes, we detected a dramatic increase in epileptiform activity with respect to wild type controls. In search for a biochemical correlate of these data, we found an abnormally high phosphorylation of Akt and ribosomal protein S6, two key controllers of neuronal circuit development and plasticity [13], which was accompanied by higher expression of vesicular glutamate transporter 2 (vGluT2). In addition, we mined the FoxG1 ChIP-Seq dataset of cortical neurons [6] to identify pathways directly regulated by FoxG1 and to validate our findings. Finally, patch-clamp recordings of synaptic activity showed an impairment in spontaneous excitatory transmission.

Our results show that the cortex of *FoxG1^+/−^* mice is affected by opposite network-wide and micro-circuit alterations, leading to higher excitability and depressed synaptic transmission, respectively. These functional alterations are paralleled by hyperactivation of signal transduction pathways linking neural activity to protein synthesis, which can contribute to excitatory/inhibitory imbalance in *FoxG1* mutant mice.

## 2. Results

### 2.1. Epileptiform Electrographic Activity in FoxG1^+/−^ Mice

**Figure 1:**
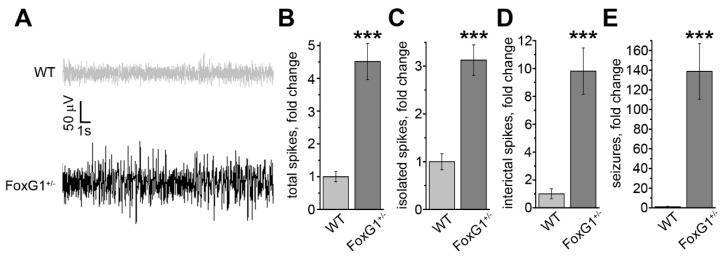
Local Field Potential (LFP) recordings of epileptiform activity in the primary motor cortex of *FoxG1^+/−^* mice. (**A**) Representative traces of LFP recordings in wild type controls (WT) and *FoxG1^+/−^* mice; (**B**) increase in the total number of high-amplitude spikes in *FoxG1^+/−^* mice (WT, *n* = 7; *FoxG1^+/−^*, *n* = 7; Student’s *t*-test, *** *p* < 0.001); (**C**) increased high-amplitude spikes in *FoxG1^+/−^* mice (WT, *n* = 7; *FoxG1^+/−^*, *n* = 7; Student’s *t*-test, *** *p* < 0.001); (**D**) increased interictal events in *FoxG1^+/−^* mice (WT, *n* = 7; *FoxG1^+/−^*, *n* = 7; Student’s *t*-test, *** *p* < 0.001); (**E**) increased number of electrographic seizures in *FoxG1^+/−^* mice (WT, *n* = 7; *FoxG1^+/−^*, *n* = 7; Student’s *t*-test, *** *p* < 0.001).

Based on our previous findings showing higher propensity to proconvulsant-induced generalized seizures [11]), we assessed the electrophysiological profile of the primary motor cortex (M1) of freely moving *FoxG1^+/−^* mice, using chronic implants for local field potential (LFP) recordings (Figure 1A). Quantitative analysis of the data revealed an overall increase in the frequency of high-amplitude spikes in *FoxG1^+/−^* animals (Figure 1B), in comparison to controls. Spiking events were then sorted according to their clustering (i.e., single vs grouped events) and duration (see Materials and Methods). A similar, significant trend could be observed for isolated spikes, interictal events, and electrographic seizures (Figure 1C–E), which were dramatically increased in comparison to wild type animals. In addition, it is important to note that isolated and interictal events were increased about 4.5- and 3-fold, whereas seizure events were 140-fold more frequent, in *FoxG1^+/−^* mice than in controls. Thus, the global increase in cortical excitability (Figure 1B) tends to boost the occurrence of long-lasting high-amplitude spiking events (Figure 1E), in comparison to single or short-lasting events (Figure 1C,D).

### 2.2. Hyperactivation of Signal Transduction and Excitation/Inhibition Imbalance in FoxG1^+/−^ Mice

**Figure 2:**
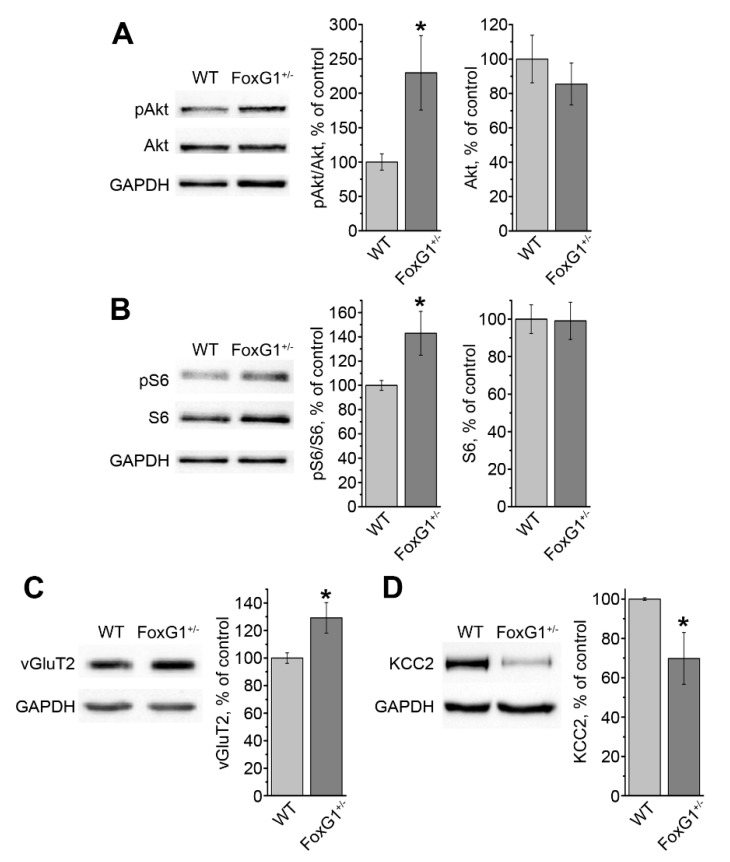
Hyperactivation of the Akt/S6 pathway and dysregulation in the excitatory/inhibitory balance in the cortex of *FoxG1^+/−^* mice. (**A**) Representative blots and quantifications showing higher Akt phosphorylation in *FoxG1^+/−^* mice compared to wild type controls (WT) (WT, *n* = 5; *FoxG1^+/−^*, *n* = 5; Student’s *t*-test, * *p* ≤ 0.05); (**B**) representative blots and quantification showing higher S6 phosphorylation in *FoxG1^+/−^* mice compared to wild type controls (WT) (WT, *n* = 5; *FoxG1^+/−^*, *n* = 5; Student’s *t*-test, * *p* ≤ 0.05); (**C**) representative blots and quantification showing higher vGluT2 expression in *FoxG1^+/−^* mice compared to wild type controls (WT) (WT, *n* = 5; *FoxG1^+/−^*, *n* = 5; Student’s *t*-test, * *p* ≤ 0.05); (**D**) representative blots and quantification showing lower KCC2 expression in *FoxG1^+/−^* mice compared to wild type controls (WT) (WT, *n* = 5; *FoxG1^+/−^*, *n* = 5; Student’s *t*-test, * *p* ≤ 0.05).

The findings from the LFP recordings prompted us to look for a biochemical correlate, which could account for the higher M1 excitability. First, we analyzed Akt activation, and detected a significant increase in phospho-Thr^308^-Akt (Figure 2A). Then, we moved downstream, following this signaling pathway, and quantified the level of phospho-Ser^235-236^S6. Consistent with Akt hyperactivation, phosphorylation of S6 was significantly higher in *FoxG1^+/−^* mice than in controls (Figure 2B).

These alterations in intracellular signaling can affect the expression of proteins controlling synaptic transmission. Therefore, we measured the levels of synaptic neurotransmitter transporters. On the one hand, expression of vesicular GABA transporter (vGAT) and vesicular glutamate transporter 1 (vGluT1) did not significantly change in *FoxG1^+/−^* mice (*FoxG1^+/−^* 95.2 ± 7.6%, WT 100.0 ± 5.8%, Student’s *t*-test, *p* = 0.629, *n* = 5 for both groups). On the other hand, expression of vesicular glutamate transporter 2 (vGluT2) was significantly higher in the M1 of *FoxG1^+/−^* mice compared to wild type animals (Figure 2C). We also quantified the expression of the potassium-chloride cotransporter, KCC2, and, consistent with our previous data on hippocampal samples [11], we found a significant decrease in its level in the M1 of *FoxG1^+/−^* mice (Figure 2D). Taken together, the above described data indicate a disruption in intracellular signaling, correlated with changes in the expression of proteins contributing to the regulation of the cortical excitation/inhibition balance.

### 2.3. FoxG1 Directly Regulates Akt Pathway Gene Network

**Figure 3:**
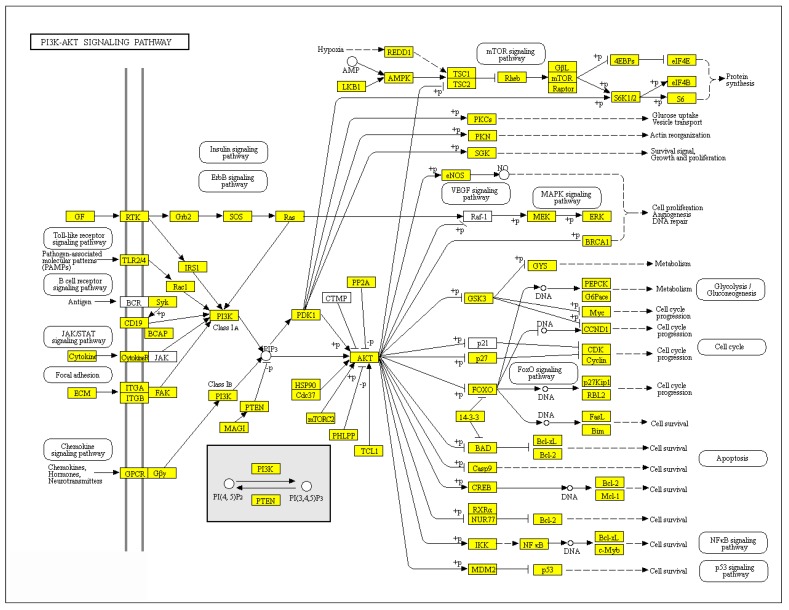
FoxG1 target genes mapped on the Akt pathway network. Known FoxG1 target genes are highlighted in yellow according to [6], and comprise Akt and S6, thus validating western blot data shown in Figure 2.

Our data demonstrate that reduced expression of *FoxG1* causes Akt hyperactivation in the mouse cortex. To determine at which level in the Akt pathway regulation FoxG1 plays a role, we analyzed its target genes, by taking advantage of the ChIP-Seq database published in [6]. This database was obtained from E15.5 mouse cortex samples i.e., when FoxG1 starts to be expressed in differentiated neurons. We classified these genes into groups of known cellular and biochemical pathways (see complete list in Supplementary Table S1), and found that most of the genes playing a role in the Akt pathway are FoxG1 target genes (Figure 3). This demonstrates a general role of FoxG1 in Akt pathway regulation and suggests that this pathway may mediate the effects of FoxG1 on cortical neuronal activity.

### 2.4. Impaired Spontaneous Excitatory Synaptic Transmission in FoxG1^+/−^ Mice

**Figure 4:**
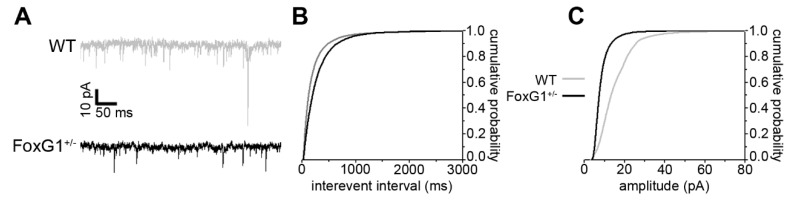
Downregulated spontaneous excitatory neurotransmission in the cortex of *FoxG1^+/−^* mice. (**A**) Representative traces from mEPSC recordings from wild type (WT) and *FoxG1^+/−^* mice; (**B**) cumulative distributions of interevent intervals showing lower mEPSC frequency in *FoxG1^+/−^* mice compared to WT controls (WT, *n* = 9; *FoxG1^+/−^*, *n* = 9; Kolmogorov–Smirnov test, *p* < 0.001); (**C**) cumulative distributions of event amplitudes showing reduced mEPSC frequency in *FoxG1^+/−^* mice compared to WT controls (WT, *n* = 9; *FoxG1^+/−^*, *n* = 9; Kolmogorov–Smirnov test, *p* < 0.001).

Taking into account the data indicating cortical macrocircuit hyperexcitability, along with altered expression of vGluT2 and KCC2, we investigated spontaneous synaptic transmission in the primary motor cortex of *FoxG1^+/−^* mice, by measuring miniature excitatory postsynaptic currents (mEPSCs) with patch-clamp recordings. Strikingly, we found that both mEPSC frequency and amplitude were decreased in *FoxG1^+/−^* mice, compared to wild type controls (Figure 4A–C).

These data indicate that, at the single-neuron level, cortical spontaneous excitatory synaptic transmission is downregulated in *FoxG1^+/−^* mice.

## 3. Discussion

Mutations in *FoxG1* have been recently recognized as causing non-X-linked Rett syndrome [7,14]. Basic neurophysiology studies on animal models can help in better understanding the consequences of this genetic alteration, and contribute to the quest for an effective therapy. In line with this, we analyzed cortical pathophysiology of *FoxG1^+/−^* haploinsufficient mice employing EEG recordings in freely moving animals. We show, in the primary motor cortex, the occurrence of spontaneous epileptiform events, which cluster into electrographic seizures with very high frequency. These results agree with our previous findings in the hippocampus of *FoxG1^+/−^* mice [11] and, altogether, they indicate an enhanced propensity to epilepsy in brain structures essential to movement and cognition.

Since epileptiform activity can trigger modifications in gene/protein expression [15] which, in turn, can increase seizure frequency and severity, we analyzed the phosphorylation of S6 ribosomal protein, which is acutely increased in rats following pentylenetetrazole (PTZ)-induced seizures [16]. The expression of phospho-S6 was higher in the cortex of *FoxG1^+/−^* mice, which indicates chronically elevated levels (no particular stimuli or treatments were delivered to the animals before sacrifice). This is in agreement with human data showing higher phospho-S6 in the cortex resected from patients affected by drug-resistant temporal lobe epilepsy [17]. Moreover, S6 phosphorylation is controlled by phospho-Akt, and higher activity of the mTOR pathway has been demonstrated in both animal models of epilepsy [18,19] and patients [17]. In keeping with this, higher phospho-Akt levels were also present in the cortex of *FoxG1^+/−^* mice. Interestingly, stronger activation of mTOR due to Akt mutation leads to cortical malformations and seizures also by disrupting FOXG1-dependent transcriptional control [20]. It is tempting to speculate that FoxG1 mutations—as observed in our experimental model—can cause hyperactivation of Akt, further impinging on FoxG1-dependent transcriptional control, thus resulting in a pathological positive feedback. Our analysis, showing that most of the genes belonging to the Akt pathway network are direct FoxG1 targets, supports this hypothesis.

Epilepsy is triggered and sustained by disruptions in the delicate balance between excitatory and inhibitory circuits [21]. Indeed, we found a higher expression of vGluT2, the glutamate transporter isoform specific of subcortical afferents [22], along with lower levels of the KCC2 potassium-chloride cotransporter, a key regulator of intracellular chloride homeostasis [23]. The latter result is consistent with our previous findings in the hippocampus [11], and with the established shift in chloride reversal potential towards more positive values contributing to circuit hyperexcitability in epilepsy [24]. On the other hand, the hippocampus of *FoxG1^+/-^* mice displays a lower expression of vesicular GABA transporter (vGAT), whereas no significant change in this protein could be detected in the cortex (*FoxG1^+/−^* 101.2 ± 14.2%, WT 100.0 ± 3.6%, Student’s *t*-test, *p* = 0.937, *n* = 5 for both groups). Taken together with the cortical increase in vGluT2, these data suggest that distinct changes in excitatory and inhibitory synapses can occur in different brain regions of *FoxG1^+/−^* mice. These different molecular alterations underlie the same macroscopic functional outcome, namely higher epileptiform EEG event frequency.

At the postsynaptic level, adult *FoxG1^+/−^* mice show lower levels of the A1 subunit of glutamate AMPA receptor (GluA1) and of PSD-95, a multifunctional organizer of excitatory synapse structure [25]. Accordingly, our patch-clamp measurements show decreased mEPSC amplitude, which can represent the electrophysiological readout of decreased density of AMPA receptors. Further work will be required to understand whether the reduction in mEPSC frequency can be ascribed to a presynaptic effect affecting neurotransmitter release, or to a global reduction in the number of excitatory synapses.

Therefore, at the micro-circuit level excitatory synaptic transmission is downregulated, in opposition to the macrocircuit level, which is characterized by higher circuit excitability and seizure propensity (current work, and [11]). This can be explained as a homeostatic plasticity manifestation [26], in which spontaneous synaptic transmission is reduced to compensate for pathologically enhanced excitability of a whole brain structure.

Our results provide evidence of opposite changes in macro- and micro-circuit excitability, accompanied by hyperactivation of Akt and S6, which can provide a functional and molecular substrate that contributes to the complex neurological phenotype of *FoxG1*-associated RTT.

## 4. Materials and Methods 

### 4.1. Animals

All mice used in this study were males generated by mating *FoxG1^+/-^* mice (a generous gift from Dr. Vania Broccoli, “Vita-Salute” University, Milan, Italy) with wild type animals of the same background (C57BL/6J, purchased from the Jackson Laboratories, Bar Harbor, ME, USA). Animals were housed in a 12 h light/dark cycle with free access to food and water. All animal procedures were approved by the Italian Ministry of Health (Decree n°258-2012/B, 23 October 2012) and were fully compliant with Italian (Ministry of Health guidelines, Legislative Decree n°26/2014) and European Union (Directive n°2010/63/UE) laws on animal research. The experimental design was set by taking into account the ARRIVE guidelines, considering the mean values of the electrophysiological, behavioral and biochemical parameters assessed, as well as their associated variances and measured variations. In addition, the principles of the Basel Declaration, including the “3R” concept, were considered throughout the whole project.

### 4.2. LFP Recordings

Electrode implants were performed at P55 by adapting the techniques described in [11,27]. Mice were anesthetized by i.p. zoletil/xylazine (80/10 mg/kg) and mounted on a stereotaxic apparatus, then the skull was exposed. A burr hole was drilled at stereotaxic coordinates corresponding to the primary motor cortex (anteroposterior −1.38 mm, mediolateral 2.00 mm to bregma) [28]. With the aid of a micromanipulator, a parallel bipolar steel electrode was positioned epidurally. A grounding screw was placed in the occipital bone. The electrodes and the reference were soldered to an electrical connector and the whole implant was secured with dental acrylic cement (Paladur, Pala, Germany). The animals were allowed to recover from anesthesia for 5 days and monitored for any sign of pain or distress during the following week. At the end of this period (corresponding to P60), the animals were placed in a recording chamber, where, after a one-hour habituation, LFP recording sessions lasted 60 min. Recordings were performed starting at 9:00 in the morning, in our case (light on at 8:00, light off at 20:00) corresponding to the tail of the active circadian phase.

The acquisition system consisted of a swivel rotor connected to an amplifier, plugged to a digitalization USB card (National Instruments, Austin, TX, USA). Signals were bandpass filtered (0.3–100 Hz) and amplified 5000 times. Analysis of number and duration of spiking events was performed by means of a LabView-based custom application, by taking the mean baseline amplitude of each signal and by establishing a threshold equal to 4.5 times its standard deviation. Every crossing of this threshold corresponded to a “spike” and spikes were considered to be clustered when separated by less than 1.5 s. Each spike group lasting more than 4 s was classified as “cluster”, whereas single spikes (i.e., with no surrounding spikes within 1.5 s) and spike groups lasting less than 4 s were considered as isolated spiking events [11,21]. To better appreciate the changes in event frequency, each individual value was divided by the corresponding WT group mean value, to obtain a normalized fold-change value.

### 4.3. Western Blotting

Following the same protocol as [11], mice were euthanized by cervical dislocation, then the brain was extracted and the hippocampi dissected out, frozen in liquid nitrogen and stored at −80 °C until further processing. RIPA buffer containing the following (in mM): NaCl 150, EDTA 5, PMSF 1, TRIS-HCl pH 7.5 10 and Triton X-100 1%, Na-deoxycholate 1%, SDS 0.1%, 1× protease inhibitor cocktail (Sigma-Aldrich, Milan, Italy) was used for protein extraction. After adding the lysis buffer, tissues were sonicated until reaching complete mechanical dissociation, then incubated for 30 min in ice and, finally, centrifuged at 20,000× *g* for 30 min at 4 °C. The supernatant was recovered and the total protein content was measured using a BSA-based Bradford assay (Bio-Rad, Hercules, CA, USA). Protein extracts (30 µg) were run on 10% acrylamide gels for 90 min at 150 V, using a TRIS-glycine buffer containing (in mM): TRIS 25, glycine 192, added with 0.1% SDS. After SDS-PAGE, proteins were transferred on nitrocellulose membranes at 250 mA for 90 min at 4 °C, using TRIS-glycine buffer added with 20% methanol. The blots were blocked for 1 h at RT under gentle rocking using 5% milk and 0.5% Tween in TBS, then incubated O/N at 4 °C under gentle rocking using primary antibody solutions prepared in the same blocking solution. The rabbit polyclonal primary antibodies used were: anti-KCC2 (1:1000, Millipore 07–432, Burlington, MA, USA), anti-phosphoThr^308^-Akt (1:1000, Cell Signaling Tech. 9275), anti-Akt (1:1000, Cell Signaling Tech. 9272), anti-phospho-Ser^235-236^-rpS6 (1:1000, Cell Signaling Tech. 2211), anti-rpS6 (1:1000, Cell Signaling Tech. 2217); GAPDH was revealed using a mouse monoclonal antibody (1:10,000, Fitzgerald Industries Int. 10R-G109a, Acton, MA, USA). Then, blots were rinsed 3 times for 10 min in TBS-Tween, and incubated with either goat anti-rabbit (1:5000, Santa Cruz Biotech. SC-2004, Dallas, TX, USA) or goat anti-mouse (1:10,000, Santa Cruz Biotech. SC-2005) HRP-conjugated secondary antibodies. The signal was revealed with ECL solutions (BioRad) and acquired using a ChemiDoc system (BioRad, Hercules, CA, USA). The optical density was quantified using the ImageJ software (NIH, Bethesda, MD, USA).

### 4.4. Pathway Analysis

Annotation of FoxG1 target genes was based on the database published in [6], and performed using the KEGG database, then visualized with Pathview.

### 4.5. Patch-Clamp Recordings

Acute slices comprising the primary motor cortex were prepared by adapting the protocol described in [29]. After sacrificing the animal by cervical dislocation, the brain was quickly extracted from the skull and submerged in ice-cold, oxygenated cutting solution containing (in mM): Sucrose 240, NaHCO_3_ 15, HEPES 10, glucose 5, KCl 2.5, CaCl_2_ 2.4, MgCl_2_ 1.2, NaH_2_PO_4_ 1.2, pH adjusted to 7.4. 300 μm thick brain sections were cut using a vibratome (Leica VT1200S, Weitzlar, Germany), then transferred to a recovery chamber filled with oxygenated artificial cerebrospinal fluid (aCSF), containing (in mM): NaCl 119, HEPES 10, glucose 10, NaHCO_3_ 6.2, KCl 2.5, CaCl_2_ 2, MgCl_2_ 1.2 NaH_2_PO_4_ 1, pH adjusted to 7.4, held at 32 °C for 30 min. After moving the chamber to room temperature and allowing 60 more min, slices were transferred to the recording chamber where recordings were performed under continuous perfusion with aCSF at 32 °C. Tetrodotoxin (0.5 μM) and picrotoxin (50 μM) were added to the bath to block the evoked synaptic activity and inhibitory synapses, respectively, in order to isolate miniature excitatory postsynaptic currents (mEPSCs). Patch-clamp recordings were performed using borosilicate glass micropipettes having a 4–6 MΩ resistance when filled with an internal solution containing (in mM): K-gluconate 145, HEPES 10, phosphocreatine 5, Mg^2+^-ATP 2.5, MgCl_2_ 2, Na^+^-GTP 0.25, EGTA 0.1, pH adjusted to 7.35 with KOH. Layer II–III pyramidal neurons were approached under DIC illumination, using a 63× immersion objective. After establishing a gigaseal, the patch was broken by applying negative pressure to achieve a whole-cell configuration. A series resistance lower than 15 MΩ was considered acceptable, and monitored constantly throughout the entire recording. At least 3 min were allowed for complete cytosol dialysis, the mEPSCs were recorded while holding the neuron at −70 mV, using a MultiClamp 700A amplifier and a Digidata 1322A card, controlled via Clampex 8.2 software (Molecular Devices, San Jose, CA, USA). Signal analysis was performed as described in [30].

### 4.6. Statistics

Statistical significance was assessed using SigmaStat 12 (SyStat Software, San Jose, CA, USA), using Student’s *t*-test. Data are expressed as mean ± SEM.

## Supplementary Materials

Supplementary materials can be found at https://www.mdpi.com/1422-0067/20/17/4127/s1.
